# Associations between resident physicians’ publications and clinical performance during residency training

**DOI:** 10.1186/s12909-016-0543-2

**Published:** 2016-01-19

**Authors:** Luke A. Seaburg, Amy T. Wang, Colin P. West, Darcy A. Reed, Andrew J. Halvorsen, Gregory Engstler, Amy S. Oxentenko, Thomas J. Beckman

**Affiliations:** College of Medicine, Department of Medicine, Mayo Clinic, 200 First Street SW, Rochester, Minnesota 55905 USA; Division of Information Services, Mayo Clinic, Rochester, MN USA

**Keywords:** Graduate medical education, Research, Clinical performance

## Abstract

**Background:**

Both research and clinical medicine requires similar attributes of efficiency, diligence and effective teamwork. Furthermore, residents must succeed at scholarship and patient care to be competitive for fellowship training. It is unknown whether research productivity among residents is related to broad measures of clinical achievement. Our goal was to examine associations between the quantity of internal medicine residents’ publications and validated measures of their knowledge, skills and multi-source evaluations of performance.

**Methods:**

This was a longitudinal study of 308 residents graduating from Mayo Clinic from 2006 to 2012. We identified peer-reviewed articles in Ovid MEDLINE between July of each resident’s match year and the end of their graduation. Outcomes included American Board of Internal Medicine (ABIM) certification examination scores, mini clinical examination (mini-CEX) scores, and validated assessments of clinical performance by resident-peers, faculty and non-physicians. Performance assessments were averaged to form an overall score ranging from 1 to 5. Associations between quantity of resident publications – and ABIM, mini-CEX and performance assessment scores – were determined using multivariate linear regression.

**Results:**

The residents published 642 papers, of which 443 (69.0 %) were research papers, 198 (30.8 %) were case reports, and 380 (59.2 %) were first-authored. On adjusted analysis, multi-source clinical performance evaluations were significantly associated (beta; 99 % CI; *p*-value) with the numbers of research articles (0.012; 0.001–0.024; 0.007), and overall publications (0.012; 0.002–0.022; 0.002).

**Conclusions:**

To our knowledge, this is the first study to demonstrate that scholarly productivity based on journal publication is associated with clinical performance during residency training. Our findings suggest that residents who invest substantial efforts in research are not compromised in their abilities to learn medicine and care for patients.

## Background

To become reflective and competent doctors, resident physicians must be trained to interpret the literature, apply evidence to patient care and demonstrate competence in research methods [1]. Therefore, the Accreditation Council for Graduate Medical Education (ACGME) requires that residents participate in scholarly activities [2].

Dedicated research time during medical school and residency is associated with increased publication by learners [3]. The Lincoln Hospital found that that 21 % of residents who participated in a research elective published, compared to only 5 % of residents who did not have the elective [4]. Likewise, a Mayo Clinic study demonstrated that internal medicine residents, all of whom engage in research rotations, publish at significantly higher rates than similar residents at other institutions [5].

Students and residents who receive dedicated research time and publish are more likely to select careers in academic medicine and ultimately achieve higher academic rank [3, 5–12]. For example, Johns Hopkins medical students with research experience were nearly three times more likely to pursue an academic medical career compared to those without such experience [9]. Similar findings have been demonstrated among resident physicians in the fields of urology [8], general surgery [6], neurosurgery [11], pediatrics [13], and emergency medicine [10].

Although research experience during medical school and residency appears to be correlated with future careers in academia, few studies have addressed potential associations between scholarly productivity and performance during residency, and research has failed to demonstrate a connection between publishing during medical school and subsequent performance during residency training [14–16]. For instance, researchers at one academic medical center found that the quantity and quality of publications during medical school had no apparent impact on future resident performance in terms of supervisory ratings and standardized test scores [17].

It remains unknown whether research productivity among resident physicians is related to broad measures of their clinical performance during residency training. Substantial time investment is required to be successful at research [18–22], which suggests that residents who excel in scholarship might suffer in their abilities to care for patients [23–25]. However, both research and clinical medicine require similar traits like efficiency, diligence, and effective teamwork, and residents must be successful at both scholarship and patient care to compete for fellowship training. Consequently, we hypothesized that there would be an association between residents’ publications, knowledge acquisition, and clinical accomplishment. Our goal was to utilize a longitudinal study design to examine potential associations between the quantity of internal medicine residents’ peer-reviewed publications and diverse measures of their clinical performance using validated measures of knowledge (ABIM certification examination scores), skills (mini-clinical examination scores) and multi-source evaluations of clinical performance.

## Methods

### Resident research curriculum

The Mayo Clinic internal medicine resident research curriculum extends across three years of training and includes the following topics: project planning, protocol design, basic principles of biostatistics and epidemiology, human subjects’ protection, and scientific writing. Educational resources are also available through the Mayo School of Graduate Medical Education, Mayo Center for Clinical and Translational Science, and biannual scientific writing workshops. Each year, residents may engage in month-long research electives, which require completion of a standardized application form, a mentor’s letter of support, and final approval by the associate program director for resident scholarship. Following their electives, residents receive evaluation and feedback from their mentors.

### Independent measure: resident publications

To quantify resident scholarship in terms of amount (counts), authorship role (first author, yes or no), and category (original research and review articles versus case report), we identified peer-reviewed articles indexed in Ovid MEDLINE between July of each resident’s match year and the end of their graduation calendar year. Articles were found by searching author last name, first initial, and middle initial when available. The resulting references were then examined manually for full-name matches. For references without full first names or where multiple authors were listed under the same name, we checked the Electronic Residency Application Service record. We excluded non-journal articles and any publication dated before July 1 of the resident’s match year. For each resident, we recorded the total number of peer-reviewed articles, case reports, and first-authored publications.

### Validity of outcome measures and co-variables

The measures used in this study are supported by validity evidence. Research has demonstrated predictive validity of the ABIM certification examination [26]. Several studies have established the validity of in-training examination (ITE) scores, including correlation with resident conference attendance and self-directed reading, and no correlation with empathy [27–29]. Validity and reliability of the mini clinical evaluation exercise (mini-CEX) has been verified by previous studies at the Mayo Clinic and elsewhere [30–36]. We use a traditional version of the mini-CEX; however, the items are on 5-point, as opposed to 9-point, scales. Clinical performance assessments of Mayo Clinic internal medicine residents are completed by peers, senior medical residents, faculty, and non-physician professionals. Validity of these assessments is based on elements from previously published instruments, input from experts with experience in scale design, factor analysis showing multiple dimensions, and excellent internal consistency reliability [37].

### Study design and data analysis

This was a longitudinal study of 308 post-graduate-year three (PGY-3) residents graduating from Mayo Clinic Rochester from 2006 to 2012. Associations of resident publications – with the outcomes of ABIM average percent correct, mini-CEX and clinical performance assessment scores – were determined using multivariate linear regression models. Scores for the clinical performance assessment items were averaged across year-three within assessor group to form an overall score ranging from 1 to 5. Co-variables adjusted for included sex (male; female), medical school (U.S. versus international medical graduate), track (categorical; clinician-investigator), PhD (yes; no), age, and percent correct on ITE examination. The threshold for statistical significance was set conservatively at 0.01 to account for multiple comparisons. This study of 308 residents provided 90 % power to detect a medium Cohen’s f^2^ effect size of 0.25 for adjusted associations between the number of publications and each outcome variable. Statistical analyses were performed using SAS version 9.3 (SAS Institute Inc., Cary, NC). The Mayo Clinic Institutional Review Board deemed this study exempt under 45 CFR 46.101, on the basis of existing data that were recorded in such a manner that the subjects cannot be identified.

## Results

Of the 308 residents, 197 (64.0 %) were male, 259 (84.1 %) were U.S. medical graduates, 279 (90.6 %) were categorical, and 11 (3.6 %) had PhD degrees. Their average age was 29.7 years. The residents published 642 papers, of which 443 (69.0 %) were research papers, 198 (30.8 %) were case reports, and 380 (59.2 %) were first-authored (Table 1).

**Table 1:** Description of the Resident Cohort (*N* = 308)

Covariate		Distribution
Gender^1^	Male	197 (64.0)
	Female	111 (36.0)
Medical School^1^	USMG	259 (84.1)
	IMG	49 (15.9)
Track^1^	CAT	279 (90.6)
	CI	29 (9.4)
PhD^1^	No	297 (96.4)
	Yes	11 (3.6)
Age at PGY3 Start^2^		29.7 (2.9)
ITE-3 % Correct^2^		72.8 (6.2)
Case Reports^3^		198 (0.6)
Research Papers^3^		443 (1.4)
First Author Publications^3^		380 (1.2)
Total Publications^3^		642 (2.1)

Notes: ^a^N (%) ^b^Mean (SD) ^c^Sum (Per Resident)

Upon adjusted analysis, multi-source evaluations of clinical performance were significantly associated (beta; 99 % CI; *p*-value) with the number of overall publications (0.012; 0.002–0.022; 0.002), and research articles (0.012; 0.001–0.024; 0.007 [Table 2]). In other words, each additional publication was associated with a 0.012-point increase in the expected mean PGY-3 multisource evaluation score after adjusting for all covariates. There were no statistically significant associations between quantity of publications and ABIM certification examination or mini-CEX scores.

**Table 2:** Associations between resident publications and measures of professionalism, clinical performance and medical knowledge

Variable	Metric^‡^	Publication type		β (CI^†^)	*p*-value
Clinical Performance	Evaluations^1^	Case Reports		0.014 (−0.007, 0.035)	0.09
		Research Papers		0.012 (0.001, 0.024)	0.007
		Combined	First Author	0.016 (−0.001, 0.032)	0.01
			Overall	0.012 (0.002, 0.022)	0.002
	Mini-CEX^2^	Case Reports		−0.008 (−0.050, 0.034)	0.62
		Research Papers		0.010 (−0.013, 0.033)	0.27
		Combined	First Author	0.007 (−0.026, 0.039)	0.59
			Overall	0.005 (−0.014, 0.025)	0.49
Medical Knowledge	ABIM^3^	Case Reports		−0.121 (−0.729, 0.486)	0.61
		Research Papers		0.125 (−0.227, 0.477)	0.36
		Combined	First Author	−0.034 (−0.529, 0.460)	0.86
			Overall	0.055 (−0.245, 0.354)	0.64

Notes: ‡ Metrics reflect average scores for PGY3 residents, except for ABIM scores which occurred after completion of residency training. † 99 % Confidence Interval (CI). ^a^Evaluations reflect overall score for multisource evaluations by resident-peers, supervisors, and allied health (scale 1–5). ^b^Mini-CEX reflects overall score (scale 1–5). ^c^ABIM reflects average percent correct

## Discussion

To our knowledge, this is the first study to explore relationships between residents’ scholarly activity in terms of publications and broad measures of their clinical performance during residency training. We found that residents’ overall numbers of publications in peer-reviewed journals were associated with validated, multi-source assessments of their clinical performance. These findings suggest that a potential overlap exists between the skills required for research and medical practice. Additionally, these findings indicate that residents who invest time in research may not be compromised in their abilities to care for patients and contribute to healthcare teams.

Although there was a positive association between resident scholarship and clinical evaluation scores, it is noteworthy that there were no negative associations between resident scholarship and any aspect of clinical performance. In other words, our findings would suggest that research does not detract from clinical training, which is the main goal of residency training [25, 38, 39]. These findings support a study of residents who, despite logging fewer clinical cases than their colleagues who did not do research, performed better on oral examinations at graduation [23]. Additionally, these findings underscore a previously-untested assumption that providing education in research during residency training likely improves residents’ skills across all of the ACGME core competencies [40].

Potential explanations for the association between resident scholarship and clinical performance include external and internal forms of motivation, as well as traits that are adaptive to both research and clinical practice [41]. As for external motivation, it is recognized that selection criteria for competitive fellowship training programs and academic appointments includes a strong emphasis on peer-reviewed publications [40, 42, 43]. Examples of internally motivating factors are intellectual curiosity and a personal drive for excellence that would translate to all aspects of medicine, including scholarship and patient care. Similarly, it has been observed that achievement in research and clinical practice require the abilities of time management, efficiency, diligence and effective teamwork [44]. It is possible that residents who are widely recognized for their research achievements enjoy a “halo effect,” which could render them attractive across diverse aspects of their work [45, 46]. Lastly, it has been shown that residents who participate in research have a higher satisfaction with residency training; this may enhance their clinical accomplishments and push them towards careers in academic medicine [47, 48].

This study expands upon previous research regarding scholarship among medical students and residents. One study did not identify a correlation between publications during medical school and subsequent in-training exam scores or global faculty-of-resident assessments during residency training [17], although this study may have been limited by low publication rates. Another study revealed that residents with research experience are more likely to pursue academic careers [9]. One study demonstrated a correlation between research experience and clinical achievement during residency [23]. However, the variables in that study were research elective and performance on an oral examination. Our study involved measured numbers of peer-reviewed publications by internal medicine residents, along with validated assessments of clinical performance, and the universally-important outcome measures of mini-CEX and ABIM certification scores.

This study has several limitations. It was conducted at a single institution, which may limit generalization of findings to other settings. Specifically, aspects of our residency research program that might be distinctive and are recognized as valuable for success include a dedicated research director, robust and longitudinal research curriculum, statistical resources, faculty with established research careers, and a strong culture of mentoring support [5, 39, 49–51]. Similarly, the assessment of clinical performance that was utilized in this study is unique to the Mayo Clinic; nonetheless, this assessment reflects the same ACGME competencies that are utilized by other institutions [2], and validity of the instruments’ scores include relations to other meaningful variables such as resident physician professionalism and well-being [52, 53].

## Conclusions

This study showed that numbers of journal publications by residents were positively associated with their clinical performance assessment scores during residency training. These findings suggest that research does not interfere with residents’ abilities to care for patients and work in healthcare teams. Potential explanations for the study findings are that certain traits – such as discipline, critical thinking ability, organization, and ability to work in teams – are adaptive to excellence in both research and clinical medicine. To be selected for fellowship programs, residents must demonstrate both scholarly and clinical success. Therefore, additional research should determine whether there is a relationship between publication during residency and future accomplishments such as selection into competitive fellowship programs.

## Abbreviations

ABIM: American board of internal medicine
ACGME: Accreditation council for graduate medical education
ITE: In-training examination
mini-CEX: Mini clinical evaluation exercise
PGY-3: Post-graduate year 3
